# Mapping groundwater-dependent vegetation in temperate climates on the example of Central Germany

**DOI:** 10.1098/rsos.260538

**Published:** 2026-10-14

**Authors:** Léonard El-Hokayem, David Emanuel Schulz, Christopher Conrad

**Affiliations:** 1 Institute of Geosciences and Geography, Martin Luther University Halle Wittenberg, Halle (Saale), Germany; 2 German Centre for Integrative Biodiversity Research (iDiv) Halle-Jena-Leipzig, Leipzig, Germany

**Keywords:** groundwater-dependent vegetation, remote sensing, biodiversity, phreatophytes, machine learning, EU Water Framework Directive

## Abstract

Groundwater-dependent ecosystems are biodiversity hotspots that provide habitat for specialized species. The European Union (EU) Water Framework Directive (WFD) stresses the importance of identifying and protecting these ecosystems. So far, most studies focus on (semi-) arid regions, where groundwater use by ecosystems is both more prevalent and easier to detect. In this study, we adapt groundwater-dependent vegetation (GDV) mapping approaches developed for dry climates to temperate regions by replacing seasonal drought-based indicators with contrasts between dry and wet years. We implement this concept by integrating for the first time to our knowledge, ECOSTRESS evapotranspiration data with high-resolution remote sensing, regional geospatial data and botanical field observations. To test our framework, we trained and validated random forest models with eight predictor variables using 166 vegetation plots to identify GDV in Central Germany. Ground-truth GDV plots prefer cooler, wetter, nutrient-richer and more acidic to alkaline environments compared to non-GDV. The final model using a phreatophyte cover threshold of 25% representing GDV plots achieved a testing accuracy of 0.97, identifying 2067 km² of GDV. Currently, only 19% is protected under the EU WFD. The proposed mapping framework offers a solution for identifying GDV in temperate regions. The new GDV maps can contribute to managing groundwater resources and preserving biodiversity hotspots in regions facing increasing droughts, ultimately supporting the implementation of the EU WFD.

## 1. Introduction

Groundwater-dependent ecosystems (GDEs) support regional biodiversity, provide refugia and stepping-stone habitats and contribute to ecosystem services such as flood mitigation, water purification and groundwater recharge [1,2]. However, declining groundwater levels owing to climate change, land-use intensification and overexploitation increasingly threaten these systems globally [3,4].

Despite their global importance and occurrence [5], GDE research remains geographically biased. Most studies have focused on arid and semi-arid biomes, where groundwater dependence is both more critical for vegetation productivity and stability and easier to detect [6,7]. In temperate regions, studies on GDE identification remain scarce, even though groundwater subsidizes tree growth and transpiration [8,9] and the overall uptake by vegetation is estimated to be 18–30% [5,10]. This spatial bias limits ecohydrological insight and hinders the development of spatially explicit management approaches for temperate GDE, as required by the European Union (EU) Water Framework Directive (WFD) [11,12].

Groundwater-dependent vegetation (GDV) comprises plant communities that rely on groundwater to sustain their composition, structure and function [13,14]. These include both groundwater-fed surface ecosystems, such as spring and wetland vegetation and phreatophyte communities accessing subsurface groundwater [14]. Phreatophytes are plant species capable of accessing groundwater that often act as ‘foundation species’ and indicators of GDV presence [15–17]. In temperate regions, GDV is typically associated with riparian zones, wetlands and groundwater-fed forests [18,19], where groundwater availability is controlled by local climate, geology and topography [20].

Direct identification of GDV can only be achieved with field-based methods, such as vegetation surveys, isotope analysis or hydrogeological sampling. However, these methods are generally resource-intensive and difficult to scale [21–23]. At the regional level, geospatial data, such as biotope types or groundwater table depth (GWTD), are often overlaid to map at least GDV potential [20,24]. Although remote sensing offers more precise and scalable approaches to mapping GDV, these have mainly been developed and tested in dry regions. [25], where the identification of persistent ‘green islands’ during seasonal droughts is widely applied [26]. Several studies suggest that such frameworks are transferable to other climates [27,28], but this transferability has never been tested. However, the absence of annual dry seasons in temperate regions complicates the detection of drought-related responses in vegetation [29]. We hypothesize that interannual contrasts between anomalously dry and wet years can provide an alternative to seasonal drought signals, allowing the key principles of arid-region GDV mapping frameworks to be transferred to temperate regions.

Evapotranspiration (ET) is a key indicator for identifying GDV as it directly reflects plant water use, drought response and ecosystem health [30]. Previous studies used MODIS or Landsat imagery together with hydrological models to estimate ET of GDV [31–33]. The ECOSTRESS mission offers ET data with higher resolution, which has been successfully applied across ecosystems [34–36]. However, despite its potential, ECOSTRESS ET data has yet to be applied in GDV mapping, representing an opportunity to improve detection and quantify water use.

In this study, we present, to our knowledge, the first high-resolution remote sensing-based framework for mapping GDV in temperate regions. Building on approaches developed for semi-arid regions (e.g. [27,37]), we adapt and extend them by integrating for the first time ECOSTRESS ET data together with Sentinel-2 Enhanced Vegetation Index (EVI), hydrogeological data and topographical predictors. Botanical field surveys of 181 vegetation plots in Saxony-Anhalt (Germany) provided ground-truth data to train and validate regional random forest models. Finally, we tested the framework for a study region in Central Germany. Existing biotope maps allowed for independent plausibility analysis of the outcomes.

Our study follows three objectives: (i) to test whether variability between anomalously dry and wet years enables the adaptation of GDV mapping frameworks from arid and semi-arid to humid temperate regions; (ii) to identify ecological and environmental indicators of GDV in humid temperate regions by integrating botanical field plots with remote sensing and geospatial data; and (iii) to produce and validate the first high-resolution GDV map for the study region of Saxony-Anhalt, Germany. By addressing these objectives, this study delivers both a novel framework for temperate GDV detection and a regional application that supports science-policy integration in groundwater and ecosystem management within the EU WFD.

## 2. Material and methods

The workflow for mapping GDV in temperate regions consisted of three analytical steps (see the supplementary material, figure S1.1 for details). First, predictor variables were derived and combined with botanical field survey data (see the supplementary material, table S1.1 for overview of datasets used in this study). Second, random forest models were trained and validated to classify GDV and generate a high-resolution map of Saxony-Anhalt in Germany. Third, model performance and the resulting GDV map were evaluated using existing biotope maps. The individual workflow components are described in the following sections.

### 2.1. Study area

Situated within the ‘temperate broadleaf & mixed forests’ biome [38], the study area encompasses the federal state of Saxony-Anhalt, Germany. The region has a warm-summer humid continental climate, with a mean annual temperature of 8.7°C and an average annual precipitation of 548 mm (1991–2021; [39]). Saxony-Anhalt lies in the Central German Dry Region, where annual precipitation can fall below 450 mm, owing to the rainshadow of the Harz Mountains, making it the driest German state. Combined with high ET, this leads to an overall negative climatic water balance [40].

Land cover in Saxony-Anhalt is dominated by intensive agriculture (56%), with forests covering 23% [41]. Most forests are even-aged monocultures of *Pinus sylvestris*, while native deciduous species, including *Quercus robur*, *Fagus sylvatica* and *Alnus glutinosa*, are restricted to fragmented remnants, often located in floodplain areas. Natural forest ecosystems currently represent only 16% of the forested area [42].

Saxony-Anhalt can be subdivided into distinct landscape units. The northern and northeastern lowlands, shaped by the Saale glaciation, feature sandy soils, pine heaths and shallow groundwater tables, such as in the Ohre-Drömling and Dumme depressions. By contrast, the south and southwest transition into mid-mountain forelands is underlain by consolidated rocks (e.g. sandstone and limestone), with generally low groundwater productivity [43]. The central lowlands are dominated by the floodplains of the Elbe, Saale and Mulde rivers, with complex alluvial and groundwater dynamics. Overall, 90% of the extracted groundwater comes from unconsolidated sandy aquifers in the north and east, probably supporting the occurrence of GDV [44].

For this study, we focused on (semi-) natural vegetation and thus excluded agricultural areas, artificial surfaces and water bodies using high-resolution regional land cover maps [45,46]. Additionally, the Harz Mountains were excluded from the study owing to a lack of data on GWTD. This may bias results towards lowland vegetation, overlooking high-elevation GDV such as mountain meadows or wetlands and limiting transferability to mountainous regions, while the absence of coastal environments further restricts represented GDV types. The final mask reduces the study area by 60% and covers 8251 km², representing vegetated landscapes across geological and climatic gradients.

### 2.2. Predictor variables

We selected eight predictor variables to represent vegetation physiology and vitality, as well as environmental controls on GDV distribution (e.g. [7,14,20,47]). These comprised four remote sensing predictors (ETdry, ETdiff, EVIdry and EVIsd) and four geospatial predictors (elevation, slope, topographic wetness index (TWI) and GWTD).

#### 2.2.1. Remote sensing predictors

Analyses were conducted for the meteorological summer (June–August), when temperate vegetation reaches maximum canopy development [48]. In contrast to frameworks for arid and semi-arid regions that rely on distinct seasonal droughts, we assessed dry-year anomalies and interannual variation by comparing vegetation vitality between dry and wet summers as identified from long-term climatic anomalies.

First, to delineate dry and wet years, we used precipitation and temperature data (daily data at 1 km resolution) from the Germany National Meteorological Service [49,50]. Anomalies were calculated relative to the 1991–2020 climate baseline for each hydrological year (September–August). Meteorological summers with highly negative precipitation anomalies were defined as dry years (2018, 2019, 2020 and 2022), while those with positive precipitation anomalies were defined as wet years (2017, 2021, 2023 and 2024; figure 1).

**Figure 1.**
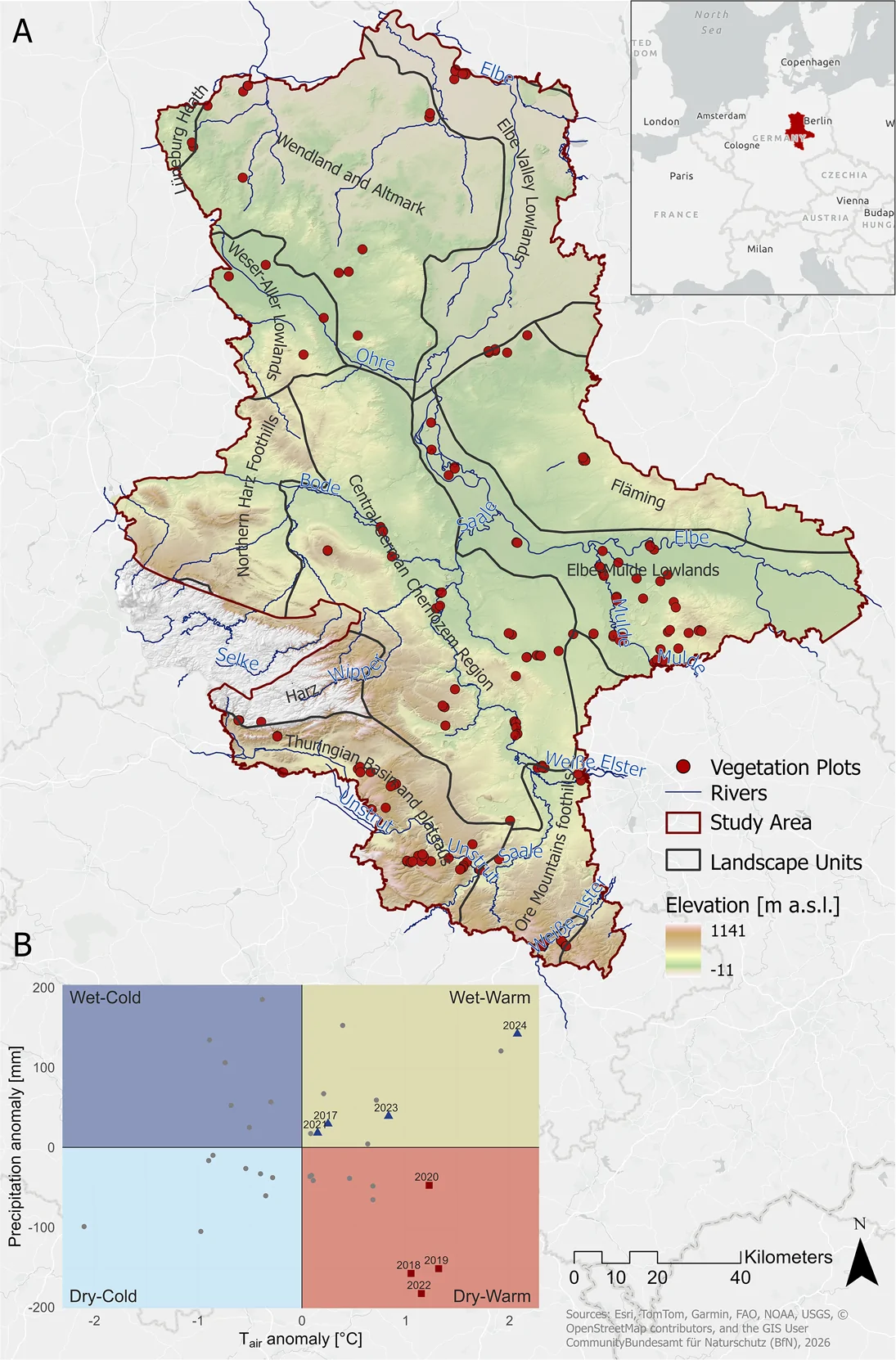
(A) Overview map of the study area Saxony-Anhalt, Germany showing the elevational gradient and the locations of the 181 vegetation surveys. (B) shows precipitation (mm) and temperature (°C) anomalies for Saxony-Anhalt calculated from Deutscher Wetter Dienst (German Meteorological Service) climate data for the reference period 1991–2020 [49,50].

Second, we integrated ET data from ECOSTRESS [51] together with vegetation indices derived from Sentinel-2 to detect GDV. ET provides a direct measure of water use by vegetation and is therefore a sensitive indicator of groundwater access, as GDV maintains high and stable ET even under drought stress [3,52,53]. ECOSTRESS retrieves land surface temperature and emissivity using a physics-based temperature and emissivity separation algorithm (TES), which forms the basis for generating higher-level products such as ET [30]. For this study, we used the Level-3 daily latent heat flux product at 70 m resolution (ECO3ETPTJPL), processed with the Priestley–Taylor Jet Propulsion Laboratory (PT-JPL) algorithm [30,54]. ET daily data were available for June–August 2018–2022, filtered for coverage (> 30% of the study area), cloud-masked and cleaned for extreme values (outside 8th–98th percentile), leaving 253 scenes for analysis (supplementary material, S1).

From this dataset, we derived two ET-based predictors to identify ‘cool and wet islands’ of GDV [13]: (i) ETdry is the mean ET during the dry summers of 2018, 2019, 2020 and 2022. It was normalized by subtracting the mean ET per land cover class [45] and dividing it by the standard deviation per class to control for vegetation-type-specific ET variation (*z*-transformation); (ii) ETdiff, the absolute difference in mean ET between the driest (2022) and wettest (2021) summers, indicating interannual stability. These predictors provide insights into water use, its stability across hydrological and climatic variation and the relative behaviour within vegetation types, providing a proxy of GDV occurrence.

Third, we quantified vegetation greenness using the EVI ([55]; equation (2.1)). EVI was calculated for 786 Sentinel-2 scenes between 2017–2024 with a spatial resolution of 10 m in Google Earth Engine [56]. Cloudy pixels were masked using the QA60 band:

(2.1) $$EVI=G\times \frac{\left(\text{NIR}\;-\;\text{red}\right)}{\left(\text{NIR}\;+\;C1\;\times \;\text{red}\;-\;C2\;\times \;\text{blue}\;+\;L\right)},$$

where NIR is near infrared and *L* is the canopy background adjustment. C1 and C2 are used to account for aerosol influences. Although the coefficients were originally developed for MODIS data, they are also widely used for Sentinel-2, as follows: *L* = 1, C1 = 6, C2 = 7.5 and *G* = 2.5 [55,57].

Finally, we calculated two vitality predictors to detect GDV following the ‘green island’ concept [26]: (i) EVIdry, the mean summer EVI across dry years, representing sustained greenness under drought; and (ii) EVIsd, the interannual variability (standard deviation) in mean summer EVI between wet and dry years. Low EVIsd indicates consistent vitality and potential groundwater access; high values suggest drought effect and hence limited access to groundwater. All pixels with mean summer EVI values less than 0.2 were excluded from the analysis as they do not represent healthy vegetation [55].

#### 2.2.2. Geospatial predictors

We included three commonly used topographical predictors to capture environmental controls on groundwater availability and consequently, GDV distribution (e.g. [20,24,47,58]). Elevation, slope and the TWI were derived from a 5m digital elevation model [59] and serve as proxies for topographically driven water accumulation [60]. The TWI was calculated in QGIS using equation (2.2) [61]:

(2.2) $$\text{TWI}=\ln\frac{As}{\tan\beta },$$

where *As* is the catchment area, and *β* is the local slope angle.

We calculated GWTD as a key control on GDV distribution [62,63] from regional groundwater contour maps [64]. Groundwater contours were interpolated into a 10 m raster surface, from which GWTD was calculated by subtracting the groundwater height from digital elevation model-derived surface elevation. Where GWTD exceeded land surface elevation, it was adjusted to the surface value. Pixels with shallow GWTD within rooting depth are more likely to contain GDV because of easier access to groundwater [8].

### 2.3. Botanical field mapping

During the summers of 2023 and 2024, we recorded 181 vegetation plots (10  × 10 m) across the study area to generate ground-truth data for spatial modelling. Area-proportional random sampling aimed to reflect the areas of hydrogeological units [65]. Yet, aquitards were under-represented owing to the applied landcover mask. For each plot, species composition, vegetation layer (tree 1: tallest trees; tree 2: trees > 5 m forming the dense canopy; shrub: 1–5 m; herb: < 1 m) and relative cover were recorded using the Braun-Blanquet cover scale [66]. Additionally, Ellenberg-type indicator values (EIV) representing light availability, temperature, soil moisture, reaction, nutrients and soil salinity [67] were assigned to each species from a European dataset [68].

Then, each recorded species was classified as obligate, facultative or non-phreatophyte, based on a global phreatophyte and groundwater-associated species checklist [69]. Since facultative phreatophytes can introduce uncertainty when used as indicators of GDV [70], site-specific refinement was necessary. Following the approach of Lenkenhoff & Rose [71], facultative phreatophytic tree species occurring on sites with GWTD above 5 m were reclassified as site-specific phreatophytes, reflecting probable groundwater access. GWTD below 5 m substantially reduces groundwater influence on tree growth and transpiration and hence indicates site-specific non-phreatophytes [8]. The ecohydrological classification for each plot was based on the site-specific phreatophyte cover (P%) as a proxy for GDV [69]. Three phreatophyte cover thresholds at 25%, 50% and 75% were evaluated to represent the proportion of the community exploiting shallow groundwater.

### 2.4. Classification of groundwater-dependent vegetation

We classified GDV and non-GDV areas using random forest, an ensemble machine learning algorithm that builds multiple decision trees from random subsets of data [72]. Random forest is widely used in remote sensing to map vegetation and groundwater-dependent ecosystems because of its robustness and capacity to model non-linear relationships [25,28,73].

As the pairwise Pearson correlation analysis was not significant, all eight predictor variables were included in the modelling (supplementary material, figure S2.1). We applied both random forest classification and regression models. For binary classification, the ground-truth vegetation plots were classified as GDV or non-GDV based on predefined phreatophyte cover thresholds. For regression, phreatophyte cover was used as a continuous response variable. A total of 15 ground-truth plots were excluded from the dataset owing to missing values for ETdry. The final ground-truth dataset consisting of 166 plots was split into 80% training and 20% testing subsets. Then, hyperparameter tuning was conducted by adjusting the number of trees, the number of variables per split, minimum leaf size, bag fraction and the maximum number of nodes hierarchically. This resulted in 108 model runs per phreatophyte cover threshold. Final model parameters were selected based on minimizing root mean square error for regression and maximizing overall accuracy for classification. The optimal random forest model was built using 50 trees, two variables per split, one minimum leaf population, 0.5 bag fraction and the maximum number of nodes available (supplementary material, table S2.1). The importance of the explanatory variables was extracted based on the Gini impurity index.

### 2.5. Plausibility analysis

We compared our mapped GDV areas to an independent biotope map [74]. This map was derived from the interpretation of colour infrared aerial orthophotos from 2009, and categorizes land cover into major units such as forest, woodland, herbaceous vegetation, water bodies, non-vegetated areas, agricultural, horticultural, viticultural and built-up areas. These units are further subdivided into 42 structural units, each representing specific biotope or land use types with associated dominant plant species.

We classified 20 structural units as likely GDV based on either their inherent groundwater reliance (e.g. swamp forests, fens, tall forbs and springs) or dominance of phreatophyte species (*Alnus glutinosa*, *Salix* spp. and *Populus* spp.). The classification, supported by literature [21,22,63,75] and expert consensus of the authors, ensured an independent reference for plausibility analysis. We compared the classified GDV maps with these reference GDV biotopes to extract true positives and false negatives and calculated producer's accuracy to quantify model performance.

## 3. Results

### 3.1. Vegetation plot analysis

The 181 vegetation surveys recorded 223 plant species, including 61 site-specific phreatophytes, of which 24 and 37 were obligate and facultative. The highest proportion of phreatophytes occurred in the first tree layer (58%), and lower proportions in the second tree layer (24%), shrub layer (26%) and herb layer (17%). The most abundant phreatophyte species in the study area are: *Fraxinus excelsior*, *Q. robur*, *Sambucus nigra*, *A. glutinosa* and *Prunus padus*.

Comparing EIV for GDV and non-GDV plots classified at the 25% phreatophyte cover threshold shows distinct ecological preferences (figure 2). While GDV plots are more often associated with moist (median moisture value = 6.6) and nutrient-rich soils (median nutrients value = 6.2), non-GDV plots indicate drier (median moisture value = 5.5) and less fertile soils (median nutrients value = 5.6). Also, while non-GDV plots are almost never found on saline soils, GDV plots can tolerate some salinity.

**Figure 2.**
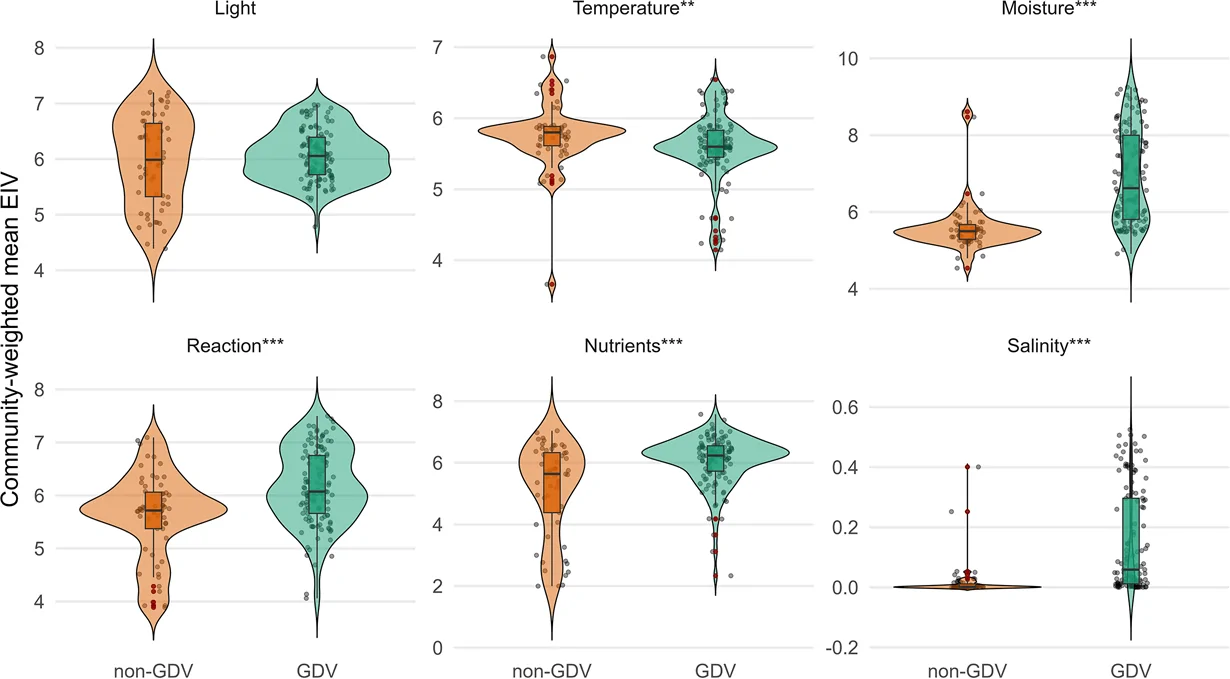
Community-weighted Ellenberg indicator values (EIV) for 181 vegetation plots from the field surveys classified as non-GDV and GDV. Wilcoxon rank-sum tests show significant differences (asterisks) between GDV and non-GDV plots for all EIV except light availability. Red dots represent outliers in the data.

### 3.2. Groundwater-dependent vegetation mapping and model evaluation

The random forest model using a phreatophyte cover threshold of 25% achieved the highest testing accuracy of 0.97. Plausibility analysis against a regional biotope map confirmed the reliability of predicted GDV areas, with 85% of GDV areas matching mapped alluvial, fen and groundwater-fed biotopes. GWTD was the most important predictor, followed by elevation, TWI and ETdiff. The random forest regression model predicts phreatophyte cover with a root mean square error of 17.4, meaning that the predicted cover is on average 17.4% higher or lower than estimated from the vegetation plots.

Approximately 2067 km² of likely GDV areas (representing 41% of all analysed pixels) were mapped across Saxony-Anhalt at 10 m resolution. Of these classified GDV areas, 52% are located within protected areas. Of these protected GDV areas, approximately one third are protected under the WFD. GDV occurrence is mainly associated with floodplain forests along the major rivers Elbe and Saale, and lowland depressions such as the ‘Ohre-Drömling.’ Large contiguous areas of non-GDV occur in upland regions such as the Harz mountains and their forelands or heaths such as the ‘Dübener Heath’ or the ‘Colbitz-Letzlinger Heath’ (figure 3).

**Figure 3.**
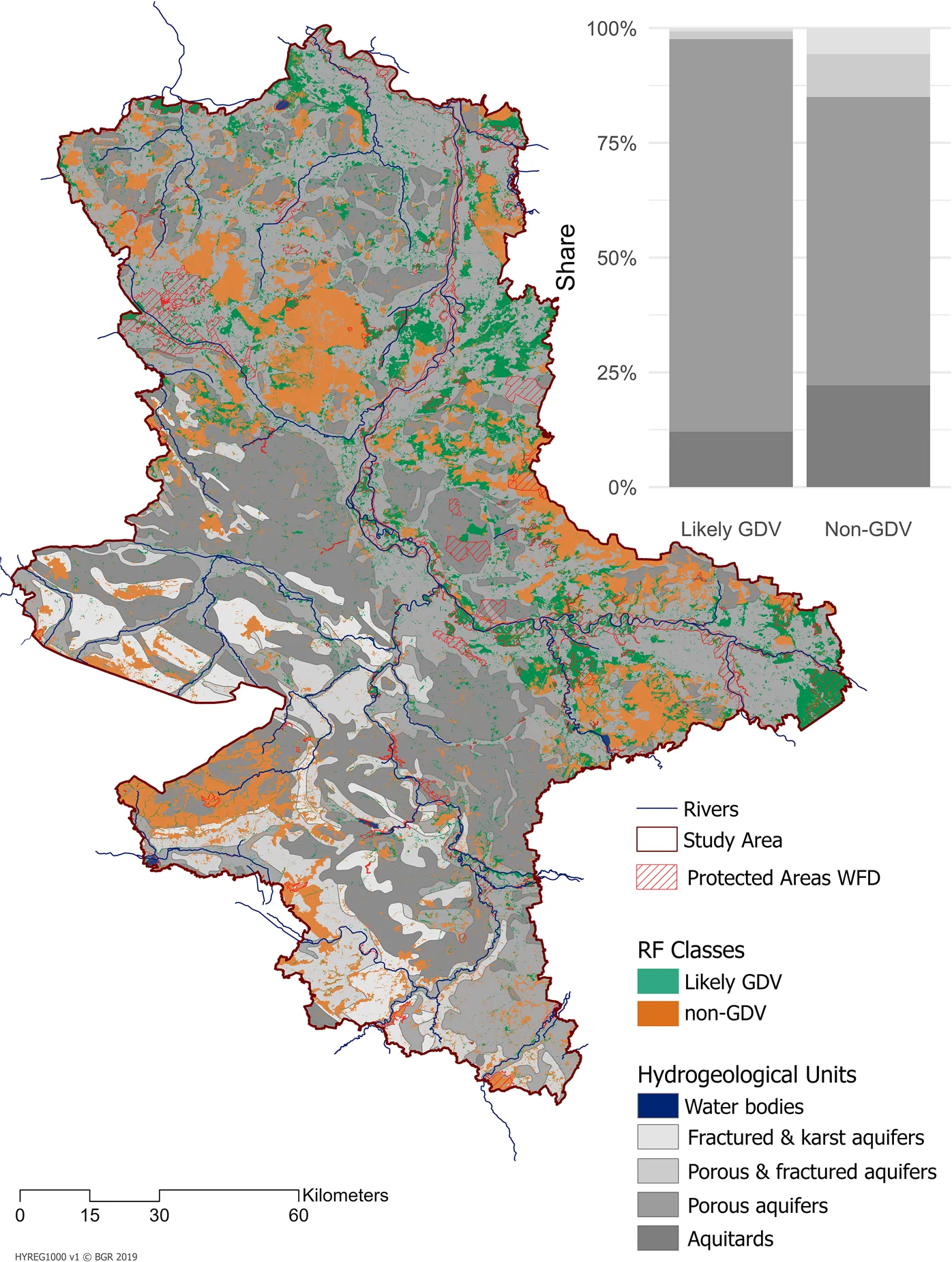
Results of the random forest (RF) classifier using the 25% phreatophyte cover threshold showing the distribution and extent of likely GDV and non-GDV in the study area and across distinct hydrogeological units [76]. The map also displays protected areas according to the EU Water Framework Directive. Alternative maps using phreatophyte thresholds of 50% and 75% and random forest regression are shown in the supplementary material, S3.

The predictor variables showed distinct spatial patterns across the study area (see the supplementary material, figure S3.1 for detailed maps). Areas classified as GDV were mainly associated with shallow GWTD and topographically wetter landscapes such as the Elbe and Mulde floodplains and northern lowlands. Remote sensing predictors showed consistent spatial patterns, with higher ETdry and EVIdry values concentrated in floodplains, lowland depressions and forested mountain foothills.

## 4. Discussion

### 4.1. Ecological patterns and management implications

Botanical field surveys revealed significant ecological differentiation between GDV and non-GDV communities. Temperate GDV favoured slightly cooler, wetter, nutrient-richer and mildly acidic to alkaline environments compared to non-GDV. However, differences in species composition and EIV for moisture and nutrients were less pronounced than those observed between semi-arid GDV and non-GDV communities in the Mediterranean biome [37]. This probably reflects the more stable hydrological regime in temperate climates, where access to soil water remains high, and there are no annual summer droughts [77]. In semi-arid and arid environments, strong seasonality and pronounced water deficits promote the dominance of deep-rooted, obligate phreatophytes such as *Banksia littoralis*, *Quercus douglasii*, *Phoenix dactylifera* or *Tamarix* spp. [14,78–80], creating clearer ecological boundaries between GDV and non-GDV. By contrast, temperate GDV in the study area are typically composed of mixed stands of facultative phreatophytes, such as *F. excelsior*, *A. glutinosa* and *Q. robur*, whose groundwater use is opportunistic and varies with location [81].

The mapped distribution of GDV in Saxony-Anhalt reflects regional hydrogeology and land use. GDV clusters predominantly along river valleys, floodplains and lowland depressions characterized by shallow groundwater and alluvial substrates, while upland areas with deep groundwater show sparse GDV occurrence. This pattern suggests that GDV occurrence is primarily driven by the accessibility of the capillary fringe, with vegetation in low-relief and glaciated northern plains maintaining a hydraulic connection to groundwater during droughts [20]. Conversely, elevated terrains and intensively cultivated loess plateaus exhibit few likely GDV areas owing to both deep groundwater and agricultural drainage [11]. A similar spatial preference of GDV into floodplain and valley systems has been observed; that is, in semi-arid Australia and South Africa [7,20,60].

The overall proportion of GDV detected in Saxony-Anhalt with 41% of (semi-) natural vegetation is comparatively high for a temperate region, where 18–30% groundwater uptake is estimated across the entire biome [5,10]. This might reflect the dry, continental climate in the study area, which may favour the development of GDV [70], as well as strong hydrological connectivity in glacial and fluvial deposits, especially in northern lowlands [82]. In the intensively used landscape of Saxony-Anhalt, only a small area of (semi-) natural vegetation remains in areas unsuitable for agriculture, such as depressions, wetlands and ravines. In turn, these landforms favour the occurrence of GDV, where they act as refugia for biodiversity and ecosystem functions under increasing climatic stress [4,60].

The observed ecological and spatial patterns highlight the azonal character of GDV, determined by groundwater availability and accessibility rather than by climatic or edaphic zonation [83]. While the species composition and ecological traits of GDV communities differ between temperate and arid regions, their distributional patterns seem comparable overall. This suggests that fundamental ecohydrological controls on GDV occurrence [6,84] might be consistent across biomes and thus comparably detectable through remote sensing and geospatial data integration [25]. Yet, mapping frameworks must be adapted to regional climatic variability and data availability.

From a management perspective, our GDV map provides spatially explicit information relevant to the EU WFD, which seeks to protect groundwater-dependent ecosystems [21]. The mapped GDV area is around 6.6 times larger than the area identified on the biotope map (313 km²). This suggests a significant amount of previously unmapped and potentially unprotected GDV. As the biotope map was created in 2009, some discrepancies may also reflect land-cover changes since its creation. Nevertheless, most mapped GDV in Saxony-Anhalt currently lie outside protected areas, indicating that these ecosystems are still under-represented in conservation planning. Linking the GDV map to groundwater body assessments could prioritize ecologically vital resources, guide abstraction limits and designate GDV sites, advancing policy integration in increasingly drought-prone temperate landscapes [11,85–87].

### 4.2. Methodological evaluation, limitations and outlook

Mapping temperate GDV is challenging owing to the absence of distinct dry seasons, which complicates the detection of drought-related vegetation responses [26]. To address this, we identified dry and wet years from precipitation anomalies, allowing interannual comparisons of vegetation vitality and ET dynamics as indicators of groundwater dependence. Our results support the transferability of the conceptual GDV mapping framework rather than the direct transfer of predictor variables developed for arid regions. However, the transferability to wetter regions needs to be further tested. Projected increases in drought frequency and severity under climate change [88,89] may improve the applicability of such approaches in temperate regions, while at the same time increasing pressure on GDV [4].

The use of satellite-derived ECOSTRESS ET data represents a novelty in GDV-mapping, providing a more direct indicator of vegetation water use. Our results show that high ET during dry conditions is a stronger predictor of GDV occurrence than interannual ET variability, suggesting that stable water use under drought is a useful signal of groundwater dependence [31,33,90]. This is consistent with findings for vegetation vitality from arid and semi-arid regions, where vegetation vitality during dry periods was more important in predicting GDV occurrence than interannual changes [28,69]. In contrast to vegetation indices from satellite data, which often show lagged responses to climate extremes up to three months [91], ET responds more directly to changes in water availability and therefore has the potential not only to indicate, but ultimately to quantify, groundwater use by vegetation [92–94]. However, forest management in the study region alters spectral signals, making groundwater detection difficult, particularly in managed pine and spruce forests. Also in such cases, ET-based indicators are advantageous as they are less sensitive to canopy disturbance. Still, high vegetation vitality does not always reflect groundwater use, since nutrient and light availability are also important controls [95].

GWTD was the most important predictor in our model, although its use in both field classification and spatial prediction requires acknowledging potential circularity. Regional patterns with shallow groundwater in northern lowlands and deeper GWTD in upland areas matched the spatial distribution of hydrogeological units and GDV, indicating that its predictive power extends beyond plot-level [82]. Excluding GWTD reduced the testing accuracy from 0.97 to 0.78, indicating that the remaining predictor variables retain predictive power, while at the same time underscoring its ecological relevance. However, this comparison is not fully independent because GWTD was also used to classify the vegetation plots where widespread facultative phreatophytes complicate classification compared to arid regions where obligate phreatophytes dominate GDV [10,70,96].

Among the evaluated phreatophyte cover thresholds, 25% yielded the highest testing accuracy. This suggests that relatively low phreatophyte cover may already indicate groundwater dependence in temperate ecosystems, where mixed communities with facultative phreatophytes contribute to groundwater use without dominating the vegetation composition. Using more conservative thresholds (50% and 75%) excludes these communities, reduces the amount of training data classified as GDV and consequently model performance. A similar optimum of 30% phreatophyte cover was reported by El-Hokayem *et al.* [69] for large-scale GDV mapping in the Mediterranean biome. This suggests that moderate phreatophyte cover provides a robust indicator of GDV occurrence across different climatic settings and vegetation.

Interestingly, variable importance changed when increasing phreatophyte cover thresholds for the ground-truth plots, indicating a transition from hydrogeological controls to functional signals of groundwater use (see the supplementary material, figure S2.2). At lower phreatophyte cover thresholds, GWTD dominated, reflecting likely GDV defined by groundwater accessibility, whereas with stricter thresholds ETdiff and ETdry became most important, suggesting that GDV with high phreatophyte coverage are characterized by differences in ET patterns rather than by groundwater availability alone. Overall, this pattern might suggest a transition from likely GDV (hydrogeological control) at low thresholds to functionally GDV (physiological control) at high thresholds. That said, conservative thresholds identify GDV by their active groundwater use rather than by environmental conditions alone, and underline the value of ET-based metrics for detecting GDV in temperate regions.

Despite methodological advances, several limitations remain for mapping GDV in temperate regions. Limited spatial coverage, coarse resolution (70 m) and short temporal record of ECOSTRESS restrict spatial transferability, fine-scale mapping and long-term analyses. Furthermore, cloud cover reduces the availability of optical Sentinel-2 and ECOSTRESS data, particularly in wet years, constraining interannual comparisons. Under warm, dry conditions, calculated ET values can exceed values in wetter years, which may cause mean ET to appear lower during cold and wet periods. Here, we focused on relative differences in vegetation water use and its stability across contrasting years, rather than absolute vegetation stress and therefore used ET anomalies and interannual differences as predictors. Additional ECOSTRESS products such as the Evaporative Stress Index, which normalizes ET to potential ET, may provide complementary information on vegetation stress, although variability in satellite overpass timing remains a challenge. Temporal inconsistencies in ECOSTRESS data availability across years (supplementary material, figure S.1.2) may introduce minor bias in ET-based predictors, although ETdiff relies on two years with comparable coverage (2021 and 2022), and ETdry represents an aggregate over multiple dry summers rather than individual events. The integration of radar data (e.g. Sentinel-1) could improve temporal coverage and provide more robust indicators of vegetation structure and plant water use in future studies [97]. A further limitation is the limited ground-truth dataset with 166 vegetation plots, which may constrain model generalization. Future studies could test the framework with reduced input data, such as habitat or biotope maps as alternative training sources. The framework was evaluated in a relatively dry humid temperate region and may require further testing across wetter temperate regions.

## Supplementary Material



## Data Availability

Data and codes (version 1.0) are openly available [98]. Supplementary material is available online [99].

## 5. Conclusion

This study presents, to our knowledge, the first high-resolution remote sensing-based framework for mapping GDV in humid temperate regions, adapting approaches developed for arid and semi-arid regions. By integrating ECOSTRESS ET and Sentinel-2 EVI, hydrogeological and topographical data with ground-truth vegetation plots, the framework achieved high classification accuracy (0.97) and produced the first regional GDV map for Saxony-Anhalt, Germany. The map shows that around 41% of the semi-natural vegetation in Saxony-Anhalt is likely to be groundwater-dependent. This is a comparatively high proportion for a temperate region, highlighting the important role that groundwater plays in sustaining vegetation under relatively dry temperate conditions. Of this area, 1754 km² were not identified on existing biotope maps. However, approximately half of these are located within protected areas, emphasizing the under-representation of GDV in current conservation frameworks. Together, these findings demonstrate that the conceptual approach to mapping GDV developed for dry regions can be transferred to temperate climates if anomalous dry years have occurred. The resulting maps provide a basis for the spatial identification and monitoring of GDV, supporting the management of groundwater, the conservation of biodiversity and the implementation of the EU WFD in temperate regions that are increasingly affected by drought.
